# Can accelerated transcranial direct current stimulation improve memory functions? An experimental, placebo-controlled study

**DOI:** 10.1016/j.heliyon.2020.e05132

**Published:** 2020-10-03

**Authors:** Martin Bystad, Benedicte Storø, Nina Gundersen, Ida Larsen Wiik, Lene Nordvang, Ole Grønli, Ingrid Daae Rasmussen, Per M. Aslaksen

**Affiliations:** a Department of Psychology, Research Group for Cognitive Neuroscience, Faculty of Health Sciences, University of Tromsø, Norway; b Department of Psychology, Faculty of Health Sciences, University of Tromsø, Norway; c Department of Child and Adolescent Psychiatry, University Hospital of North Norway, Norway; d Department of Geropsychiatry, University Hospital of North Norway, Norway

**Keywords:** Neuroscience, Cognitive neuroscience, Nervous system, Cognition, Learning and memory, Memory, Transcranial direct current stimulation, Neuropsychology, Neuromodulation, Cognitive enhancer

## Abstract

The aim of this study was to investigate whether transcranial Direct Current Stimulation (tDCS) could improve verbal memory functions in healthy old and younger participants. We hypothesized that active tDCS led to significantly improved memory function, compared to placebo tDCS. Forty healthy participants (20 old and 20 younger participants) were included in the study. We applied a novel stimulation protocol, where six sessions of anodal tDCS were administrated during two consecutive days. Each tDCS session lasted 30 min. The current intensity was 2mA and the stimulation area was the left temporal lobe at T3 in the 10–20 EEG system. Immediate recall, delayed recall and recognition memory were assessed with California Verbal Learning Test II (CVLT-II) and executive functions were assessed with the Trail Making Test (TMT) before the first tDCS session and after the last tDCS session. Half of the participants received placebo tDCS, whereas the other half received active tDCS. We did not reveal any significant differences between active and placebo tDCS in memory functions. However, there was a significant difference between active and placebo tDCS in executive function measured by the Trail Making Test (TMT). This experimental study failed to reveal significant differences between active and placebo accelerated tDCS for verbal memory functions. However, accelerated tDCS was found to be well-tolerated in this study.

## 1 Introduction

A method that may improve memory functions in healthy individuals is called transcranial direct current stimulation (tDCS) (Manenti et al., 2013). This is a non-invasive stimulation method aimed to enhance plasticity and learning (Prehn and Flöel, 2015). tDCS treatment is performed by placing two or more electrodes on the scalp (one stimulation electrode and one reference electrode). The position of the stimulation electrode depends on the cortical area targeted for stimulation. Then, a weak current (2 mA or less) is delivered through the stimulation electrode. tDCS is simple to administer and it is associated with few adverse effects (Brunoni et al., 2012).

tDCS works by modulation of cortical excitability and neuroplasticity (Nitsche and Paulus, 2001). Thus, tDCS aims to increase neuroplasticity through the process of long-term potentiation (LTP) (Monte-Silva et al., 2013). This involves an increase in synaptic strength and is crucial for neuroplasticity and memory (Lynch, 2004). tDCS does not directly cause neuronal firing, but trigger conditions that makes neuronal firing more likely (Reinhart et al., 2017).

Previous studies suggest that tDCS may enhance memory functions (Prehn & Flöel, 2015). Memory improvement from tDCS could be due to enhanced excitability in the temporal cortex (Boggio et al., 2012; Ferrucci et al., 2008). Some studies also suggest that tDCS may enhance memory consolidation by affecting resting state networks and brain-wave frequency (e.g Annarumma et al., 2018; Kirov et al., 2009; Marshall et al., 2011).

Sandrini and colleagues (Sandrini et al., 2014) found that a 15 min active tDCS session could significantly improve recall of a wordlist after 30 days. Furthermore, another study found that tDCS could improve verbal memory functions in both old and young participants (Manenti et al., 2013). Prehn and colleagues found that a combination of selective serotonin reuptake inhibitor (SSRI) and tDCS could give significant better immediate memory in both younger and older participants (Prehn et al., 2017). However, no such effects were found for delayed recall.

Verbal memory functions decline with age (Cargin et al., 2007). Thus, it could be assumed that aging can affect the efficacy of tDCS, when tDCS is used as a memory enhancer. For instance, Ross and colleagues found that tDCS stimulation of the temporal lobe could improve name recall for faces in both younger and older participants (Ross et al., 2011). However, older participants improved more compared to younger participants. One assumption is that that aging weakens cortical connections and that tDCS may enhance neuronal firing in a higher degree than for younger participants (Gutchess, 2014). tDCS may work better for old, since younger individuals have a nearly optimal level of neuroplasticity and thus smaller potential for improvement. However, a recent study (Leach et al., 2018) found that younger participants improved more than older participants and that older participants may be less receptive to tDCS. It is uncertain whether older participants benefit more from tDCS than younger participants. Hence, there is a need to investigate if the effect of tDCS differs between old and younger individuals.

It is also found that tDCS can improve memory functions in patients with Alzheimer's disease. Boggio and colleagues (2012) found that 30 min sessions of active tDCS for five consecutive days could lead to a nearly 10 % improvement in recognition memory. This improvement was prolonged for one month and was significantly higher in patients who underwent tDCS than in those who received placebo tDCS, which only led to a 2.6 % improvement.

On the other hand, Bystad and colleagues (2016a) found no significant differences in memory improvement between active and placebo tDCS in patients with Alzheimer's disease. In Alzheimer's disease, studies using tDCS have shown inconsistent results (Kim, 2016). In healthy individuals, tDCS is also associated with mixed results (Tremblay et al., 2014).

Before tDCS can be validated as a therapeutic tool, it is important to investigate different stimulation protocols in healthy individuals, in order to find the optimal stimulation protocol. In addition, since cognitive functions are relevant for our function in daily life it can be useful to investigate if tDCS leads to cognitive improvement.

The optimal number of tDCS sessions and the interval between sessions remain uncertain (Woods et al., 2016). For both experimental and clinical application of tDCS, the lack of standardized protocols possesses a problem when conducting new studies or comparing results between studies (Cappon et al., 2016).

It is assumed that a high repetition rate, with short intervals between each tDCS sessions can probably be more efficient than increasing the duration of the stimulation (Nitsche et al., 2015; Woods et al., 2016). Such high repetition rate may lead to longer lasting effects, since the neurophysiological after-effects of tDCS is relatively short lived. For instance, a recent study suggested that 13 min of tDCS stimulations of 2mA leads to 90 min after-effect (Thair et al., 2017).

To prolong the effect of tDCS, it has been proposed to use short intervals (<30 min) between sessions (Woods et al., 2016). Such short intervals between each session can be referred to as “accelerated tDCS” (Bystad, Rasmussen, Abeler and Aslaksen, 2016). A previous case study found that such application of tDCS could improve memory functions in patients with early stage Alzheimer's disease (Bystad et al., 2016b). However, to date, this protocol has limited evidence.

Based on previous studies (Manenti et al., 2013; Sandrini et al., 2014), we aimed to investigate the effect of accelerated tDCS on memory functions and executive functions in both healthy old and healthy younger participants. We applied an accelerated tDCS protocol, with short (30 min) intervals between each session. We hypothesized that active tDCS would lead to a significantly improved verbal memory function (immediate recall, delayed recall and recognition), compared to placebo tDCS.

## 2 Materials and methods

### 2.1 Participants

A total of 40 individuals participated in the study. There were 20 old (59–69 years, mean age = 63 years, 16 females) and 20 young (19–30 years, mean age = 22 years, 13 females) participants. The eligibility criteria were absence of any serious somatic or psychiatric conditions or injuries to the central nervous system that could impact cognitive functions. Such conditions included cancer, cerebrovascular diseases, chronic obstructive pulmonary disease, heart failure, depression/anxiety and psychosis. All participants completed the Hospital Anxiety and Depression Scale (HADS) (Mykletun et al., 2001), a questionnaire used to screen for depression and anxiety.

Patients with scores above 15 on the HADS were excluded because depression may affect cognitive functions (Lam et al., 2014).

### 2.2 Recruitment

Participants were recruited by advertisement. All participants were informed that the experiment aimed to investigate if tDCS could improve memory functions. The study was executed in a research laboratory at the University of Tromsø, Department of Psychology. All participants signed a written informed consent prior to participation. They were compensated with a gift-card, worth 500 NOK (approximately 59 USD) after the participation. The study was approved by the Regional Ethical Committee for Research Ethics in Medicine and Health Sciences (2012/1890).

### 2.3 Outcome measures

In the present study, the primary outcome measure was verbal memory functions, assessed with the California Verbal Learning Test–Second Edition (CVLT-II) (Delis et al., 2004). The CVLT-II is a standardized neuropsychological test, normalized by age and gender. The CVLT-II assess immediate recall, delayed recall and recognition. The CVLT-II is widely used (Delis et al., 2004) and based on list-recall, where the participant is instructed to recall a list with 16 words. The CVLT-II has good test-retest reliability (Delis et al., 2004). To reduce test-retest practice effects, we used the standard version at baseline, and the alternative form after the last tDCS session. The standard and alternative forms have different word-lists.

The secondary outcome measures included the Trail Making Test A and B (TMT A and TMT B) (Tombaugh, 2004) and the Digit Span test from the Wechsler Memory Scale (WMS) (Wechsler, 1998). TMT A measures sustained attention, speed and motor function, whereas TMT B also assesses executive functions. It should be noted that neither the TMT A or TMT B are memory tests. The TMT A consists of 25 circles spread over a paper. These circles have numbers from 1-25. The participant is instructed to draw lines to connect the circles from 1 to 25 in ascending order and try to do this a fast as possible. The TMT B consists of circles (1–13) and letters (A-L). The participant is instructed to draw lines as fast as possible to connect the circles in ascending and alternating order between letters and numbers (i.e 1-A-2-B-3-C-4-D, etc.). WMS Digit Span measures attention/working memory. The participant is instructed to repeat a cumulative sequence of numbers forward and backward.

To control for general cognitive abilities, the Matrix Reasoning and Vocabulary tests from the Wechsler Abbreviated Scale of Intelligence (WASI) (Pearson, 1999) were conducted at baseline. To screen for cognitive impairment among the old participants we used the Mini Mental Status Evaluation (MMSE-NR) (Folstein et al., 1975).

To assess possible adverse effects, we used a questionnaire from Brunoni and colleagues that was translated into Norwegian (Brunoni et al., 2011). This questionnaire asks specifically about adverse effects from the tDCS procedure, specifically regarding itching, tingling, headache and discomfort (Brunoni et al., 2011).

### 2.4 Transcranial direct current stimulation (tDCS)

The stimulation was delivered using a direct current NeuroConn stimulator (neuroConn, Ilmenau, Germany). This is a rechargeable battery driven device that terminates the stimulation if the voltage exceeds safety limits or if the impedance is too high. The impedance level was kept below 10kΩ. The stimulation duration was 30 min and the current intensity was 2mA. The current was transferred to the skull through a pair of 35-cm2 rubber electrodes. To improve connection on the electrode-scalp interface we used a Ten20 neurodiagnostic electrode paste (Weaver and Company, Colorado, USA). The anode (stimulation electrode) was placed at the T3 position in the 10–20 system (a system used for electroencephalographic electrode positioning). This positioning was applied in previous studies (e.g Boggio et al., 2012) and is recommended for memory improvement in Alzheimer's disease (Zhao et al., 2017). We wanted to target the temporal lobe and found it reasonable to assume that Zhao and collegaues (2017) recommendations should be relevant for healthy participants. The left temporal cortex plays a major role in verbal memory (Frisk and Milner, 1990; Johnson et al., 2001), hence, we wanted to target this area. The cathode (reference electrode) was placed at the Fp2 position, i.e on the right frontal lobe.

All participants were assigned their own five-digit code. This code determined if the tDCS device should give the placebo or active stimulation. Neither the experimenter nor the participant knew if the tDCS stimulator delivered the active or placebo stimulation. Thus, the study was double blind. The order of the codes was randomized using the Random.org website (https://www.random.org/).

Prior to the study, we did not ask the participants about their expectations or attitude to tDCS. The electrode placement and sessions duration were identical for active and placebo tDCS. In the beginning of the placebo tDCS session, a current was delivered for 30 s and then there was a “ramp-down” procedure that faded the current automatically.

### 2.5 Procedure

Participants met individually for two consecutive days in a research laboratory at the university. First, each participant received information about the study. Then, the participant underwent the memory assessment. The duration of this assessment was approximately 60 min. The immediate and delayed recall task from CVLT-II was administrated at the beginning of the assessment. When the assessment was completed, the first tDCS session began. Three sessions were given on both the first day and the second day. Each tDCS session lasted for 30 min. The break between the sessions was about 30 min. After the final tDCS session, the participant underwent memory assessment. Here, the immediate and delayed recall task from CVLT-II was also administrated at the beginning of the assessment. The cognitive tests were not presented in a counterbalanced order, since the CVLT-II has parallel versions.

**Figure 1:**
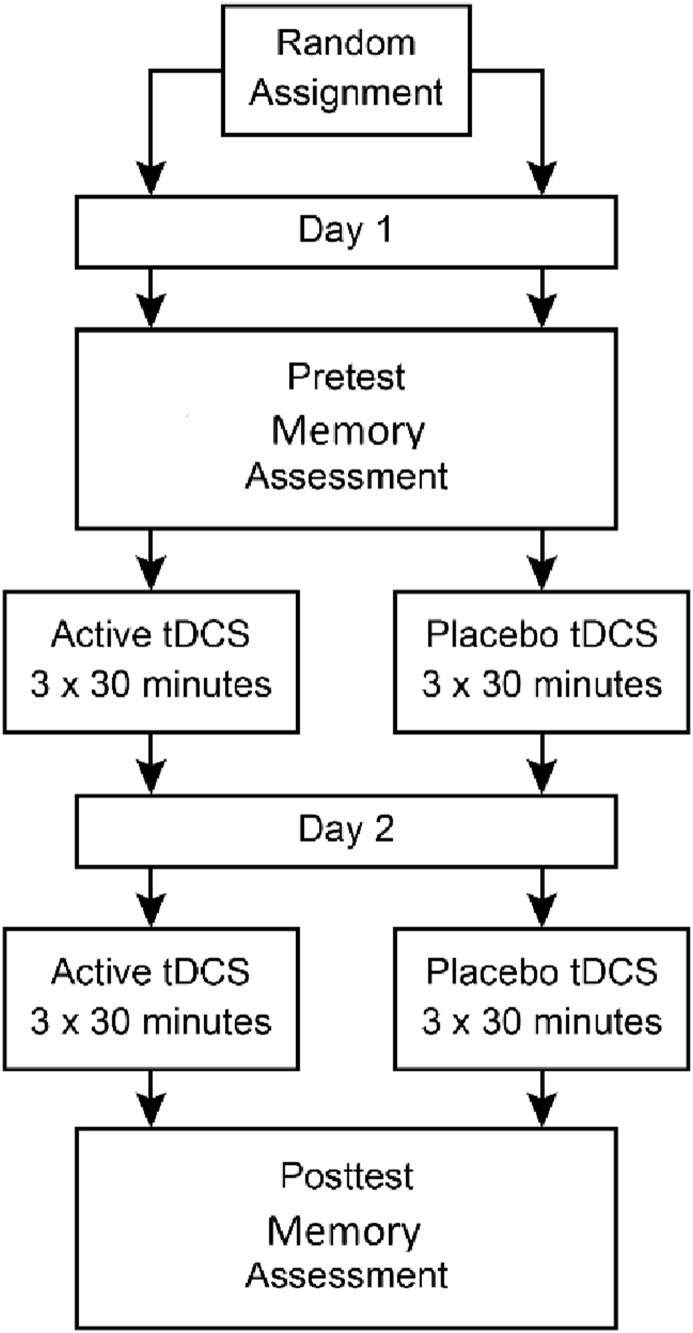

See Figure 1 for an overview of the procedure.

### 2.6 Statistical and power analysis

All data were analyzed in SPSS Version 24 We calculated the change scores between baseline and post stimulation memory assessment scores to investigate the effect of the tDCS stimulation. We conducted independent t-tests to investigate the differences in the mean change of scores between placebo and active tDCS. A Multivariate Analysis of Variance (MANOVA) was conducted to investigate group differences between placebo and active tDCS adjusted for age. Data were normally distributed, shown by Shapiro Wilk test.

A previous study (Sandrini et al., 2014) with healthy participants found that active tDCS led to significant improvement in verbal memory functions, compared to placebo tDCS. In that study, tDCS was delivered only once, with a 15-minute duration. Based on mean scores from Sandrini et al. (2014), we used a power estimation calculator (clincalc.com) and estimated that our study had 80 % power in order to achieve a significant effect with a least 32 participants (16 placebo and 16 active tDCS). Thus, we wanted to include a total of 40 participants. The alpha-level was 0.05.

## 3 Results

Table 1 displays the number of participants who improved on different outcome scores from baseline to post-test. The analysis showed no significant differences in CVLT-II scores between the active and the placebo tDCS (Table 2). For CVLT-II immediate recall F = 0.067, df = (1.0), *p* = 0.79, CVLT-II delayed recall F = 0.24, df = (1.0), *p* = 0.62 and CVLT-II recognition F = 0.092, df = (1.0), *p* = 0.76, no significant differences were found between the active and the placebo tDCS. However, we found that the active group scored significantly better on change scores than the placebo group on TMT-B, F = 4.54, df = (1.0), p = 0.040.

**Table 1:** Frequency table.

	Active tDCS (N = 20)	Placebo tDCS (N = 20)
CVLT-II immediate recall	9	6
CVLT-II delayed recall	4	4
CVLT-II recognition	5	3
TMT-A	17	13
TMT-B	18	18
Digit Span	11	17

The data represent the number of patients who showed improvements (>) on the outcome measures.

Improvement was considered as improved scores from baseline to the post-test.

**Table 2:** Changes in cognitive scores for all participants (N = 40).

Outcome	Group		Age Group		Age Group ∗ Group	
	F	*p*	F	*P*	F	*p*
CVLT immediate	0.67	0.797	0.14	0.70	0.32	0.57
CVLT delayed	0.24	0.622	3.00	0.092	0.544	0.46
CVLT recogntion	0.092	0.20	0.39	0.535	0.25	0.61
TMT-A	3.02	0.91	3.81	0.59	0.007	0.93
TMT-B	4.54	0.040	0.18	0.66	0.33	0.97
Digit Span	0.84	0.365	0.77	0.78	0.055	0.81

Note: “Group” is active or placebo, “Age Group” is younger or old and “Age Group ∗ Group” is the interaction between the group and the age group. A MANOVA analysis was conducted.

For the group of old participants, we found no differences in the CVLT-II scores between active and placebo tDCS (see Table 3). For CVLT-II immediate recall *t* (15.69) = - 0.90, *p* = 0.37, CVLT-II delayed recall *t* (14.83) = 0.18 *p* = 0.85 and CVLT-II recognition *t* (11.19) = 0.43, *p* = 0.67, no significant difference were found between active and placebo tDCS. For TMT A *t* (17.94) = 2.02, *p* = 0.058 and TMT B *t* (15.92) = 0.64, *p* = 0.52, and Digit Span *t* (12.45) = - 0.98, *p* = 0.91, there was no significant difference between active and placebo group.

**Table 3:** Change in scores for old participants (N = 20).

	Group	Mean	Std. Deviation	*P value*	*Hedges g*
CVLT immediate	Placebo	-4.90	10.98	0.37	0.38
	Active	-1.10	7.34		
CVLT delayed	Placebo	-0.35	0.97	0.85	0.07
	Active	-0.43	1.01		
CVLT recognition	Placebo	-0.25	0.58	0.67	0.18
	Active	-0.44	1.23		
TMT-A	Placebo	-2.10	7.50	0.058	0.86
	Active	-9.10	7.93		
TMT-B	Placebo	-13.80	28.05	0.52	0.28
	Active	-21.25	20.66		
Digit Span	Placebo	10.20	1.47	0.91	0.04
	Active	10.10	2.51		

Note: The mean values are the estimated change from baseline to post-testing (post testing minus baseline). For the CVLT-II immediate recall score, the mean value is displayed as a T-score. For the CVLT-II delayed recall and recognition scores, the mean value are displayed as Z-scores. An independent t-test was applied to calculate the differences between the placebo and active tDCS groups. For the CVLT scores and Digit Span scores, a positive values indicates a positive change. For TMT A and B, negative values indicate improvements.

For the group of younger participants, we found no difference in CVLT-II scores between active and placebo tDCS (see Table 4). For CVLT-II immediate recall *t* (18.00) = 0.22, *p* = 0.82, CVLT-II delayed recall *t* (17.69) = - 0.82, *p* = 0.42 and CVLT-II recognition *t* (17.82) = - 0.58, *p* = 0.56, and no significant difference were found between active and placebo tDCS. For TMT-A *t* (17.99) = 1.08, *p* = 0.29 and Digit Span *t* (16.90) = - 0.48, *p* = 0.63 there were no significant differences between the active and placebo groups. However, on TMT-B *t* (11.47) = 3.26, *p* = 0.007, the active group scored significantly better than the placebo group.

**Table 4:** Changes in scores for younger participants (N = 20).

Outcome	Group	Mean	Std. Deviation	*P* value	*Hedges g*
CVLT immediate	Placebo	-4.30	9.80	0.82	0.09
	Active	-5.30	9.83		
CVLT delayed	Placebo	-1.25	1.29	0.42	0.35
	Active	-0.80	1.13		
CVLT recognition	Placebo	-0.60	0.90	0.56	0.26
	Active	-0.35	1.00		
TMT-A	Placebo	-8.00	9.92	0.29	0.46
	Active	-12.80	9.79		
TMT-B	Placebo	-10.20	6.90	0.007∗	1.39
	Active	-30.50	18.43		
Digit Span	Placebo	0.50	2.06	0.63	0.20
	Active	0.90	1.59		

### 3.1 Adverse-effects

No adverse-effects were reported, neither in young participants or old participants, based on a questionnaire (Brunoni et al., 2011) for adverse-effects in tDCS procedures.

## 4 Discussion

The aim of the present study was to investigate whether accelerated tDCS could improve verbal memory functions in healthy young and healthy old participants. We also investigated whether tDCS could affect executive functions in both young and old participants. In addition, we wanted to study if age was a significant factor of tDCS efficacy.

We did not reveal significant differences between placebo and active tDCS in verbal memory functions. This was not in accord with results from two previous studies (Manenti et al., 2013; Sandrini et al., 2014). Furthermore, we did not find any significant differences in verbal memory between placebo and active tDCS, whilst adjusting for age.

However, we found a significant difference between placebo and active tDCS for executive functions, as measured with TMT-B. This significant difference was only found among the younger participants. It should be noted that none of our participants reported any adverse effects, despite the short intervals between each the tDCS sessions. Accelerated tDCS seems to be both safe and well-tolerated in our study, based on the questionnaire (Brunoni et al., 2011) from our participants.

The reason for our non-significant effect of tDCS on verbal memory functions may be attributed to several different causes. First, we applied a novel stimulation protocol (i.e., accelerated tDCS, with short intervals between each session). To our knowledge, no studies have investigated such an intensive protocol. Accelerated tDCS is based on recommendations from Nitsche and colleagues (2015), rather than evidence. This protocol may not be as efficient as we expected.

Second, it also uncertain if tDCS actually leads to cognitive (Horvath, Forte and Carter, 2015b) and neurophysiological (Horvath, Forte and Carter, 2015a) changes in healthy individuals. Horvath and colleagues argue that tDCS has some major shortcomings (e.g., electric current influences, inter-subject variability) (Horvath et al., 2014). For instance, Tremblay (2014) reported that one participant experienced a 251 % increase in motor evoked potentials, whereas another participant experienced a 41 % decrease. Anatomic differences (e.g skull thickness) and neurophysiology are individual factors that may affect the distribution of current flow to the cortex (Horvath et al., 2014). The effect of tDCS on cognitive function in healthy participants is associated with conflicting results (Tremblay et al., 2014). Such intra-individual sensitivity to tDCS may be an obstacle in pursuing its effect on cognition. Thus, it is uncertain whether the between subject design is the most appropriate choice for investigation of effects of tDCS on cognitive function.

Hsu and colleagues (2015) argues that tDCS may work best in pathological states and benefit those who need it most, since there may be a ceiling effect in healthy participants and tDCS may serve to strength weaken pathological neural circuits. Consequently, we cannot disregard a lack of effect from our tDCS stimulation.

We revealed a significant effect on executive functions in the young participants. A possible explanation is the limitation of the TMT-B test, which was used to measure executive functions. TMT-B seems to have substantial test-retest practice effects, especially over a short interval (Bartels et al., 2010). For instance, a study (Bartels et al., 2010) found that retest with TMT-B after three weeks could improve the score with nearly 10 s. Since memory test usually have parallel versions, TMT-A and TMT-B are more susceptible to test-retest practice effects. According to our results (Table 1), most participants improved on the TMT-B test, regardless of placebo or active tDCS. We cannot completely rule out a test-retest practice effect.

Further, tDCS has low specificity (Csifcsak et al., 2018). Even if our aim was to stimulate temporal cortex, other cortical areas may also have been affected (e.g frontal cortex), since tDCS may lead to a widespread alterations of functional connectivity (Keeser et al., 2011). It is suggested that tDCS may enhance alerting attention (Coffman et al., 2012) and affecting the resting state networks (e.g Annarumma et al., 2018; Kirov et al., 2009) This could improve executive function, and thus lead to better scores on TMT-B. Effects of tDCS on resting state connectivity may be different in younger adults (Prehn and Flöel, 2015; Woods et al., 2019).

### 4.1 Limitation

The present study has several limitations that needs to be addressed. One limitation is that we relied solely on cognitive functions for our outcome measures. Consequently, we do not know if the tDCS stimulation induced any neurophysiological changes. There may be a chance that our tDCS protocol affected neuroplasticity and neural activity. However, this remains unknown in our study.

Further, a second limitation is our “one size fits all” approach. It is reasonable to assume that anatomical differences (e.g., skull thickness) can affect the efficacy of the tDCS stimulation, i.e how the current is distributed to the cortex. We did not apply a computational model to calibrate the tDCS stimulation for each participant. Our lack of individual calibration is a limitation, since individual differences can be an important factor (Sarkar et al., 2014).

A third limitation is that we did not combine tDCS with any cognitive stimulation. We only applied tDCS. This could affect the efficacy of our tDCS protocol, since the effect of tDCS may improve when tDCS and cognitive stimulation are used simultaneously (Hsu et al., 2015).

### 4.2 Future research

Further research should take advantage of both memory/cognitive assessment and psychophysiological measures (e.g., event-related potentials or neuroimaging). A combination of such outcome measures will provide insight into the cognitive and neurophysiological effect of tDCS. There is clearly a need to investigate the potential effect of tDCS on neurobiological changes in healthy individuals. For future research, it can also be useful to calibrate the tDCS procedure for each participant. A computation model can be applied in order to determine out how individual differences will affect the current distribution. In addition, it could be of potential interest to study the effect when tDCS and cognitive stimulation are delivered simultaneously.

### 4.3 Conclusions

This experimental study did not reveal a significant difference between active and placebo accelerated tDCS for verbal memory functions. However, we found that the tDCS stimulation led to a significant improvement in executive function in younger participants, assessed with TMT-B. Our accelerated tDCS protocol, with short intervals between each session, was well tolerated with no side effects of the stimulation. Future research should combine memory/cognitive and neurophysiological outcome measures.

## Declarations

### Author contribution statement

M. Bystad: Conceived and designed the experiments; Analyzed and interpreted the data; Contributed reagents, materials, analysis tools or data; Wrote the paper.

N. Gundersen, B. Storø, I.L. Wiik, L.Nordvang: Performed the experiments; Contributed reagents, materials, analysis tools or data; Wrote the paper.

O. Grønli I.D. Rasmussen: Analyzed and interpreted the data; Wrote the paper.

P.M. Aslaksen: Conceived and designed the experiments; Analyzed and interpreted the data; Wrote the paper.

### Funding statement

This research did not receive any specific grant from funding agencies in the public, commercial, or not-for-profit sectors.

### Competing interest statement

The authors declare no conflict of interest.

### Additional information

No additional information is available for this paper.
